# *Phlomidis Radix* Extract Alleviates Paclitaxel-Induced Neuropathic Pain by Modulating Spinal TRPV1 in Mice

**DOI:** 10.3390/plants12223819

**Published:** 2023-11-10

**Authors:** Keun-Tae Park, Seong-Gyu Ko, Woojin Kim

**Affiliations:** 1 Department of Physiology, College of Korean Medicine, Kyung Hee University, Seoul 02453, Republic of Korea; cerex@naver.com; 2 Korean Medicine-Based Drug Repositioning Cancer Research Center, College of Korean Medicine, Kyung Hee University, Seoul 02447, Republic of Korea; epiko@khu.ac.kr

**Keywords:** CIPN, paclitaxel, *Phlomidis radix*, TRPV1

## Abstract

Paclitaxel is a chemotherapeutic drug reported to have excellent activity against tumors; however, various side effects, including peripheral neuropathy, limit its use in some cases. In this study, the effect of *Phlomidis radix* (*P.Radix*) extract was assessed on paclitaxel-induced cold and mechanical peripheral neuropathy in mice. Multiple paclitaxel injections (accumulative dose of 8 mg/kg, i.p.) induced increased behavioral responses to cold and mechanical stimuli in mice from D10 to D21 after the first paclitaxel injection. Cold and mechanical stimuli were performed by acetone drop and von Frey filament, respectively. Oral administrations of 25% ethanol extract of *P.Radix* (300 and 500 mg/kg) relieved cold and mechanical pain in a dose-dependent manner. Furthermore, among the various transient receptor potential (TRP) cation channel subfamilies, paclitaxel upregulated the spinal gene expression of transient receptor potential vanilloid 1 (*TRPV1*) and melastatin 4 (*TRPM4*), but not ankyrin 1 (*TRPA1*). However, 500 mg/kg but not 300 mg/kg of *P.Radix* extract significantly downregulated the gene expression of *TRPV1* but not *TRPM4*. Among the components of *P.Radix*, sesamoside was identified and quantified by high-performance liquid chromatography (HPLC), and the administration of sesamoside (7.5 mg/kg, i.p.) showed a similar analgesic effect to 300 mg/kg *P.Radix*. These results suggest that *P.Radix* and sesamoside should be considered when treating paclitaxel-induced neuropathic pain.

## 1. Introduction

Paclitaxel, first isolated from *Taxux brevifolia*, is an antitumor drug used for the treatment of lung, ovarian, and breast cancers [1,2,3]. It inhibits the division of cancer cells by increasing the stability of microtubules [4]. However, according to the pharmaco-toxicological profile of paclitaxel, it is characterized by various side effects such as allergic reaction, bone marrow suppression, hair loss, and pulmonary inflammation. Among these side effects, acute and chronic peripheral neuropathy are one of the most severe side effects. Peripheral neuropathy is especially characterized as cold and mechanical dysesthesia, showing abnormal responses to cold and mechanical stimuli in the treated patients. These severe peripheral neuropathies can also interrupt the treatment schedule and limit the dosage used [5,6,7]. Therefore, there is a great need for the development of new drugs that could effectively alleviate paclitaxel-induced neuropathic pain.

Transient receptor potential (TRP) cation channels were first reported in *Drosophila melanogaster* in the *Drosophila* family in 1989 [8]. Furthermore, in the central and peripheral nervous system (CNS and PNS, respectively), TRP channels are found in various neural cell types that participate in the generation and processing of pain signals [9,10]. TRP channels were first focused for its role on temperature sensors [11,12]. Subsequently, various TRP channel subfamilies have been discovered along with their diverse functions, such as mediators of calcium release [13].

Among the TRP subfamilies, transient receptor potential cation channel subfamily V member 1 (TRPV1) is well reported for its role in pain [14]. TRPV1 is known to respond to stimuli such as capsaicin, a major component of pepper [15,16,17]. However, it is not only activated by capsaicin, but also by heat (>43 °C), acidosis, and various lipids [18]. The subfamily of TRPV consists of six families (TRPV1–TRPV6), and the most studied channel is TRPV1 [19,20]. TRPV1 senses noxious chemical agents and physical stimuli, and is involved in various physiological process, making it a prospective target for pain relief drugs [21]. Since several years, numerous bioavailable TRPV1 antagonists have been studied, and they demonstrate that modulating the function of TRPV1 could be used as a target to treat diseases, including the pain [22,23,24]. Transient receptor potential melastatin 4 (TRPM4) is also a member of the TRP subfamily. Unlike other Ca^2+^ permeable TRP channels, it is a Ca^2+^-impermeable channel and is activated only by intracellular Ca^2+^ increase [25]. TRPM4 is only permeable to monovalent cations, and Na^+^ and K^+^ are the most permeable. TRPM4 is activated by temperatures ranging from 15 °C to 35 °C degrees [26], and it is directly gated by intracellular calcium and are found in the kidneys, osteophytes, liver, heart, and spleen [25,27]. Transient receptor potential cation ankyrin 1 (TRPA1) was considered a noxious cold-sensing channel because it is mostly expressed in a subset of small neurons and was activated at temperature below 17 °C [11,28]. Serval studies have reported its involvement in pain, especially cold allodynia or hyperalgesia [29,30]. It is a bradykinin receptor that is dependent and activated by bradykinin, potentially functioning as a signal interrater very similar to TRPV1 [31].

*Phlomidis Radix* (*P.Radix*) is the dried roots of *Phlomis umbrosa* Turczaninow (Turcz.) and is one of the most widely used herbal medicines in Korea. It is widely distributed in China and Korea. Several studies have reported on chemical constituents of *P.Radix* [32,33]. High-performance liquid chromatography (HPLC) analysis identified sesamoside, shanzhiside methylester, 8-O-acetylshanzhiside methylester, and umbroside as the main components [34,35]. Although the number animal studies conducted with *P.Radix* is small, published studies have mostly focused on its role in bone formation [36,37]. However, its role in pain, especially chemotherapy-induced neuropathic pain, has never been studied before.

Thus, in this study, first, the effect of ethanol extract of *P.Radix* on paclitaxel-induced neuropathic pain was assessed. Second, the changes in the spinal TRP families (i.e., TRPV1, TRPM4, and TRPA1) were evaluated by quantifying their encoding gene expressions. Third, the effect of *P.Radix* extract on spinal TRPV1 gene and protein expressions was measured. Finally, sesamoside, the major component of *P.Radix*, was identified and quantified in *P.Radix* extract, and its analgesic effect was also investigated.

## 2. Materials and Methods

### 2.1. Animals

Male 6-Week-old C57BL/6 mice purchased from Daehan Bio (Chungbuk, Republic of Korea) and housed under controlled conditions (12 h light/12 h dark cycles, 23 ± 2 °C, 65 ± 5% humidity). Mice were acclimated for more than one week prior to all experiments. The mice were provided with a rodent standard diet (Purina, Sungnam, Republic of Korea) and water ad libitum. All animal experimental protocols were used in strict accordance with the guidelines by the Kyung-Hee University Animal Care and Committee (KHUASP-23-223, 12 April 2023).

### 2.2. Phlomidis Radix (P.Radix) Extract Preparation

Dried *P.Radix* was provided by Allborn (Pocheon, Republic of Korea). *P.Radix* was extracted using the reflux method with 25% ethanol for 6 h at 80 °C. The extract was filtered and concentrated by decompression at 60 °C. *P.Radix* was dried using an evaporator and lyophilized by a freeze dryer. The *P.Radix* extract was diluted in distilled phosphate-buffered saline (PBS) and administered orally at each concentration in a constant volume of 0.1 mL. Specimen No’ KWJ-0002.

### 2.3. Paclitaxel, P.Radix, and Sesamoside Administration

Paclitaxel (Sigma Aldrich, St. Louis, MO, USA) was dissolved in 50% ethanol and 50% Cremophor EL (Sigma Aldrich, St. Louis, MO, USA) solution to a concentration of 6 mg/mL. Prior to intraperitoneal (i.p.) injection, paclitaxel was diluted in PBS to obtain a final concentration of 0.2 mg/mL. The vehicle group was injected with the same volume of ethanol and Cremophor EL solution via the same route. Paclitaxel and vehicle were injected intraperitoneally every other day for four days (day 0, 2, 4, and 6). A total of 8 mg/kg of paclitaxel was injected to induce thermal and mechanical peripheral neuropathy in mice. The *P.Radix* extracts were administered orally via gavage using oral sonde (Jungdo-BNP, Seoul, Republic of Korea) to mice at different doses of 100, 300, and 500 mg/ kg in PBS. Sesamoside was administered to mice intraperitoneally or orally at concentrations of 5 mg/kg and 7.5 mg/kg.

### 2.4. Behavioral Tests

Acetone drop tests and von Frey filament tests were conducted to assess cold and mechanical pain in mice. Acetone drop test was assessed by spraying 10 μL of acetone onto the mice’s paw without touching the skin. In brief, mice were acclimated to the chamber for 30 min before assessment. An acetone drop was sprayed using a pipette to the hind paw. The withdrawal, licking, and flinch response time was recorded within 30 s. In the Result figures, the ‘# of Response’ refers to the average number of frequencies in each measurement [38,39].

Von Frey filament test was performed by using the von Frey filaments. Filaments with different stiffness (0.02; 0.04; 0.07; 0.16; 0.4; 0.6; 1; 1.4; and 2 g) were pierced into the mid plantar hind paw. The ‘50% threshold value’ was calculated according to the up–down method of Dixon’s and Chaplan’s calculation [40,41]. After the assessment, mice were anesthetized with isoflurane and euthanized via PBS intracardiac perfusion for tissue sampling. The lumbar 4–5 spinal cord segments were collected for further experiments.

### 2.5. Quantitative Real-Time Polymerase Chain Reaction (qRT-PCR)

Total ribonucleic acid (RNA) was isolated from the spinal cord and extracted by using an AccuPrep RNA Extraction Kit (Bioneer, Daejeon, Republic of Korea), according to the protocol. The concentration of RNA was quantified using a NanoDrop spectrophotometer (Thermo Scientific, Middlesex, MA, USA). Complementary DNA (cDNA) synthesis was performed using Maxime RT Premix (Intronbi, Seongnam, Republic of Korea). The qRT-PCR was performed by using a SensiFAST SYBR kit (Meridian Bioscience, Cincinnati, OH, USA) and the CFX Real-Time PCR System (Bio-rad, Hercules, CA, USA). The primer used for the PCR were as follows: transient receptor potential vanilloid 1 (*Trpv1*) forward 5′- GGC TGT CTT CAT CAT CCT GCT GCT-3′ and reverse 5′- GTT CTT GCT CTC CTG TGC GAT CTTGT-3′; transient receptor potential cation channel subfamily M member 4 (*Trpm4*) forward 5′-CCC TGA GGA TGG TGT TGA GT-3′ and reverse 5′- AGG AGC ACT GGG ATG TCA AT-3′; transient receptor potential cation channel subfamily A member 1 (*Trpa1*) forward 5′-CCT GCT TCA CAG AGC CTC GTT ATT-3′ and reverse 5′- GCC TAC AGG CAT AAT GGA GAG GTG-3′; glyceraldehyde 3-phosphate dehydrogenase (Gapdh) forward 5′-GTC GTG GAG TCT ACT GGT GTC TTC-‘3′ and reverse 5′-GTC ATC ATA CTT GGC AGG TTT CTC-3′. The reaction was preheated for 5 min at 95 °C followed by 40 cycles at 94 °C for 20 s, 57 °C for 20 s, and 72 °C for 20 s. GAPDH were used to standardize the amount of RNA in each sample. The qRT-PCR data for amplification-produced fluorescence were used to calculate a specific detection threshold (Ct value). Relative gene expression was quantified based on the average Ct value for each gene and the same amount of RNA (0.2 μg). The relative quantification of each group was calculated using the delta delta Ct (ΔΔCt) method, and the expression value of the control group was expressed as 1.

### 2.6. Western Blot

Spinal tissues were lysed using radioimmunoprecipitation assay buffer (RIPA) buffer and centrifuged at 13,000 rpm for 10 min. The protein assay was performed using a Bradford protein assay kit (Bio-Rad Laboratories, Hercules, CA, USA). Samples were then loading to 10% sodium dodecyl sulfate polyacrylamide gel electrophoresis (SDS-PAGE) and transferred to a nitrocellulose membrane at 120 V for 90 min. The transferred membrane was blocked with 5% skim milk for 1 h. After, the membranes were washed with 0.5% tris-buffered saline with tween 20 (TBS-T) and incubated with primary antibodies overnight in 4 °C. TRPV1 and actin were detected with rabbit polyclonal anti-TRPV1 (dilution 1:1000, #PA1-183, Invitrogen, Carsbad, CA, USA) and rabbit polyclonal anti-actin antibody (dilution 1:1000, #NB100-1617, Novus Biologicals, Littleton, CO, USA), respectively. They were further incubated with secondary antibody (horseradish-conjugated goat anti-rabbit IgG at a 1:5000, #31460, Thermo scientific, Waltham, MA, USA) for 1 h at room temperature. Bounded antibodies were then detected using an ECL solution (D-Plus ECL Femto System, Republic of Korea).

### 2.7. Identification and Quantification of Sesamoside in P.Radix Extract

The chemical compound of the *P.Radix* extract was injected and analyzed using an Agilent 1260 HPLC equipped with an ultraviolet detector (Agilent, Wilmington, DE, USA). The condition for the sesamoside standard and the *P.Radix* analysis are listed in Table 1. A stock solution of sesamoside was prepared in ethanol. The sesamoside standard used in this study was purchased from Chemface (Wuhan, China). Five dilutions of sesamoside (6.25, 12.5, 25, 50, and 100 ug/mL) were prepared and subjected to an analysis. 100 mg of the *P.Radix* sample was ultrasonically extracted (4 °C, 30 min) using 1 mL of ethanol. The diluted solution was centrifuged (4 °C, 10,000 rpm, and 30 min), and the supernatant was filtered using a 0.45 um syringe filter to obtain a test solution (Table 1).

## 3. Results

### 3.1. Multiple Paclitaxel Injections Induce Increased Responses to Cold and Mechanical Stimuli in Mice

**Figure 1:**
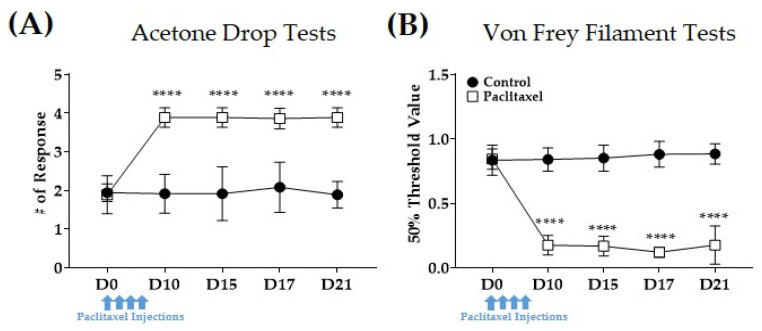
Effect of multiple paclitaxel injections on acetone drop and von Frey filament tests in mice. Intraperitoneal injections of 2 mg/kg of paclitaxel were performed every two days starting from day 0 (D0) until day 8 (D8). The accumulative dose of paclitaxel was 8 mg/kg. Behavioral assessments were conducted on D0, D10, D15, D17, and D21. Acetone drop and von Frey filament tests were conducted to assess changes in behavioral responses to cold (**A**) and mechanical (**B**) stimuli, respectively. Control group receives 50% ethanol and 50% cremophor EL solution to a concentration of 6 mg/mL diluted in phosphate-buffered saline (PBS) to a final concentration of 0.2 mg/mL. N = 6 for each group; **** *p* vs. Control group with a two-way ANOVA followed by Tukey’s post-test for multiple comparisons.

As reported in our previous studies, multiples paclitaxel injections significantly induced pain in mice. Paclitaxel was injected four times (D0, D2, D4, and D6) intraperitoneally [42]. Control group mice received the solvent of paclitaxel (i.e., 50% ethanol and 50% Cremophor EL solution to a concentration of 6 mg/mL diluted in PBS to a final concentration of 0.2 mg/mL). Acetone drop and von Frey filament tests were conducted to assess cold and mechanical hypersensitivity in mice. The results show that multiple paclitaxel injections induce increased responses to cold stimuli (Figure 1A) and a decreased threshold to mechanical stimuli (Figure 1B). Subsequently, most of the experiments were conducted on D15–D17 when pain was significantly induced in mice.

### 3.2. Analgesic Effect of Single Administration of Phlomidis Radix Extract in Cold and Mechanical Peripheral Neuropathy-Induced by Paclitaxel

**Figure 2:**
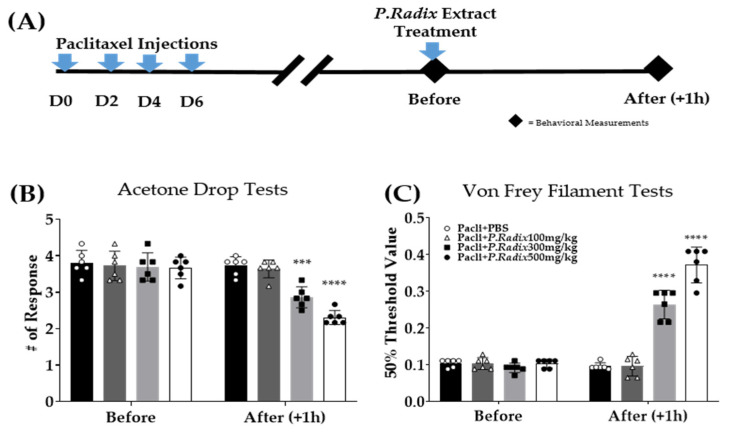
Effect of oral administration of *P.Radix* extracts on acetone drop (**B**) and von Frey filament (**C**) tests. The experimental schedule (**A**). All group received 8 mg/kg of paclitaxel (i.p.). Behavioral assessments were performed before (before) and 1 h after (After (+1 h)) the oral administration of 100, 300, and 500 mg/kg of *P.Radix* extracts or PBS. PBS was used as control to *P.Radix*. *P.Radix* or PBS was given orally to mice. N = 6 per group. *** *p* < 0.001, **** *p* < 0.0001 vs. Pacli+PBS with two-way ANOVA followed by Tukey’s multiple comparisons test.

Three different doses of *phlomidis radix* (*P.Radix*) extracts (100, 300, and 500 mg/kg) were administered orally to paclitaxel-injected mice to determine whether *P.Radix* could alleviate paclitaxel-induced neuropathic pain. Behavioral tests were performed on D15 (15 days after the starting of paclitaxel injections (Figure 1)), when significant changes to acetone drop and von Frey filament stimuli were induced (Figure 2A). Acetone drop and von Frey filament tests were used to assess cold (Figure 2B) and mechanical (Figure 2C) hypersensitivity in mice, respectively. Behavioral changes were observed before and 1 h after *P.Radix* extract administrations in mice. The results show that 300 and 500 mg/kg, but not 100 mg/kg of *P.Radix* extracts alleviate both types of pain induced by paclitaxel.

### 3.3. Gene Expression of TRPV1 and TRPM4, but Not TPRA1, Are Downregulated after Multiple Paclitaxel Injections

**Figure 3:**
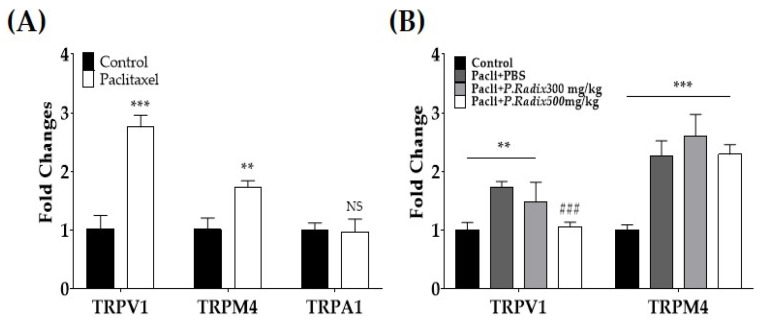
Changes of gene expressions of transient receptor potential vanilloid 1 (*TRPV1*), melastatin 4 (*TRPM4*), and ankyrin 1 (*TRPA1*) in the spinal cord following paclitaxel (**A**) and *P.Radix* extract (**B**) administrations. The gene expressions were assessed by using quantitative real-time polymerase chain reaction (qRT-PCR). Spinal cords of lumbar 4–6 level were harvested for gene analysis in paclitaxel- and *P.Radix*-extract-injected mice. Experiments were conducted on D15 after paclitaxel injection. N = 6 per group. NS, non-significant; ** *p* < 0.01, *** *p* < 0.001, vs. Control; ### *p* < 0.001 vs. Pacli+PBS with student’s *t*-test (**A**), with one-way ANOVA followed by Tukey’s multiple comparisons tests (**B**).

Gene expressions of *TRPV1*, *TRPM*, and *TRPA1* were analyzed in the spinal cords of paclitaxel injected mice. The lumbar 4–5 spinal cord segments were collected for quantitative real-time polymerase chain reaction (qRT-PCR). The results show that spinal *TRPV1* and *TRPM4* gene expressions increased by about to 154.4% and 7.06% in the paclitaxell group compared to the vehicle-treated group (Control) mice, whereas there was no significant difference in *TRPA1* (Figure 3A). As only the relative gene expression of *TRPV1* and *TRPM4* significantly upregulated in paclitaxel treated group mice, in the next experiment, the expression of *TRPV1* and *TRPM4*, but not *TRPA1*, were assessed after *P.*Radix (300 and 500 mg/kg, p.o.) extract treatments (Figure 3B). *P.Radix* extract dose-dependently modulated the gene of *TRPV1* but not *TRPM4*. The effect was statistically significant only in the 500 mg/kg treated group (Figure 3B).

### 3.4. The Protein Expression of Spinal TRPV1 Is Downregulated after P.Radix Extract Treatment

**Figure 4:**
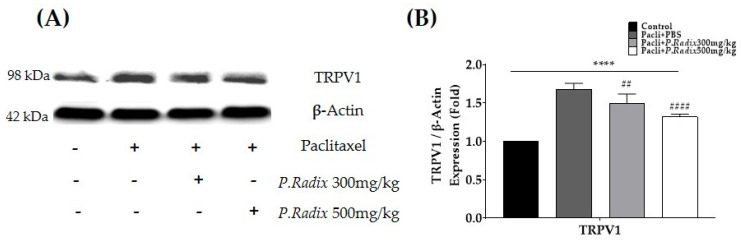
Protein quantification of spinal TRPV1 after *P.Radix* extract administrations in paclitaxel−induced peripheral neuropathic pain mice. Protein was assessed by using Western blot. Spinal cords of lumbar 4−6 level were harvested for Western blot analysis. A representative image of Western blot (**A**) and analyzed relative fold levels of TRPV1 protein in the spinal cord (**B**). All group except the control group mice received 8 mg/kg of paclitaxel (i.p.). *P.Radix* extract treated group received 300 mg/kg or 500 mg/kg of P.Radix (p.o.). PBS was used as a control to *P.Radix*. N = 6 per group. **** *p* < 0.0001, vs. Control; ## *p* < 0.01, #### *p* < 0.0001 with one-way ANOVA followed by Tukey’s multiple comparisons tests.

As the gene expression of spinal TRPV1 upregulated and downregulated following paclitaxel and *P.Radix* extract treatments, respectively, Western blot was performed to assess whether the change in gene expression is associated with that of protein in the spinal cord (Figure 4A,B). The lumbar 4–5 spinal cord segments were collected in all groups mice. Indeed, the results show that paclitaxel injections (Pacli+PBS) upregulated TRPV1 protein expression by up to 68.1% compared to control, whereas both 300 and 500 mg/kg of *P.Radix* extract administrations downregulated the increased expression of TRPV1 (11.3% and 22.0%, respectively). Although in qRT-PCR experiments 300 mg/kg did not induce significant changes, in Western blot, 300 mg/kg downregulated the protein expression as 500 mg/kg.

### 3.5. Identification and Quantification of Sesamoside in P.Radix Extract by Using High-Performance Liquid Chromatography (HPLC)

**Figure 5:**
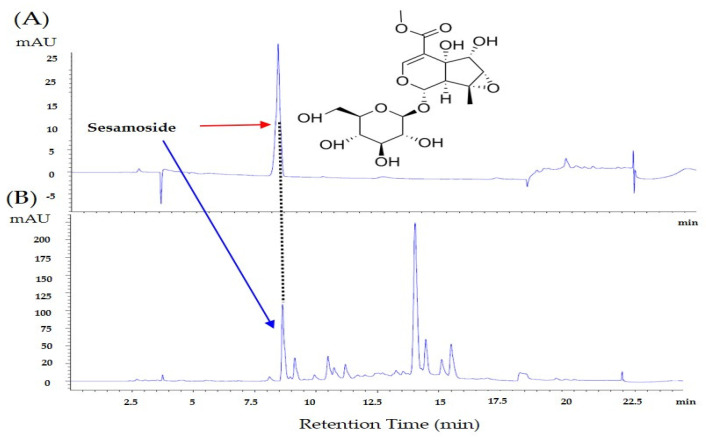
Identification and quantification of sesamoside in *P.Radix* extract by using HPLC. HPLC chromatograms of standard (**A**) and sesamoside (**B**). Arrows in the figure represent the detection curve of standard (red arrow) and sesamoside (blue arrow). The numbers in the X−axis refers to the retention time (min), whereas in the Y−axis, it represents the milli-absorbance units (m-AU).

High-performance liquid chromatography (HPLC) analysis was performed to identify and quantify, sesamoside as the active ingredient in *P.Radix* extract. The retention time of sesamoside was about 8.6 min in the standard solution (Figure 5A), and the retention time and UV spectrum of sesamoside in *P.Radix* extract solution were consistent to that of the standard (Figure 5B). Calibration curve shows linearity within the sesamoside detection range (100, 50, 25, 12.5, and 6.25 μg/mL). The sesamoside regression equation was y = 12.85621 + 3.71301 and RSQ = 0.99991. The content of sesamoside in *P.Radix* extract is shown to be approximately 1.56%.

### 3.6. Sesamoside Alleviates Paclitaxel-Induced Cold and Mechanical Hypersensitivity in Mice

**Figure 6:**
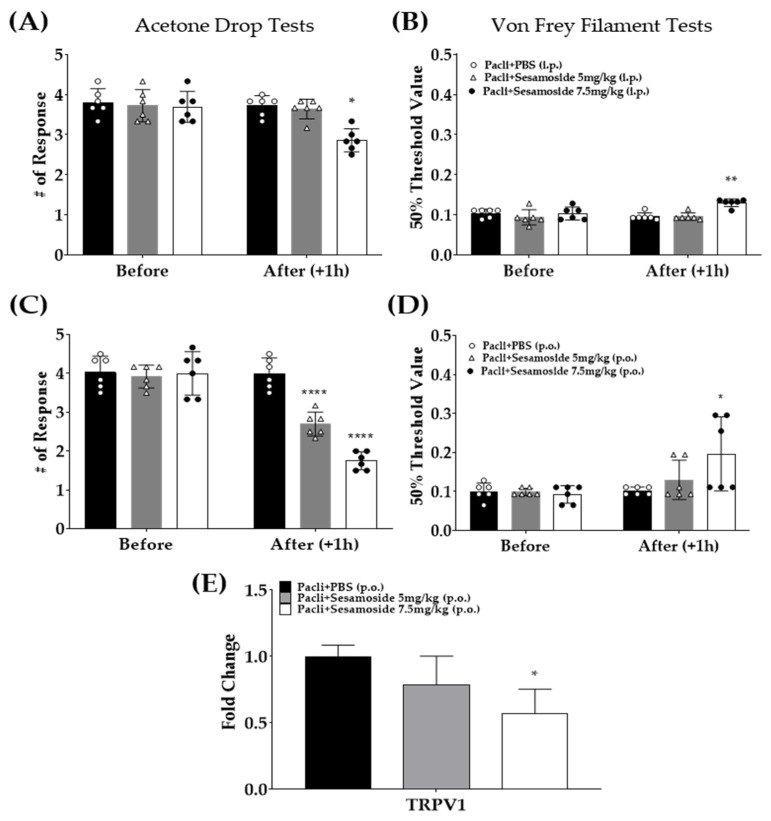
Effect of sesamosides on peripheral neuropathy induced by multiple-paclitaxel injections in mice (**A**–**D**). All group mice received 8 mg/kg of paclitaxel (i.p.) to induce cold and mechanical hypersensitivity in mice. Two different doses of sesamoside (5 and 7.5 mg/kg) were administered intraperitoneally (**A**,**B**) or orally (**C**,**D**) to mice. Changes of gene expressions of transient receptor potential vanilloid 1 (*TRPV1*) in the spinal cord following sesamoside oral administrations were assessed (**E**). Acetone drop and von Frey filament tests were performed to assess cold and mechanical hypersensitivity in mice. Behavioral tests were evaluated twice, before (Before) and after (After (+1 h)) sesamoside administrations. The gene expressions were assessed by using quantitative real-time polymerase chain reaction (qRT-PCR). Spinal cords of lumbar 4–6 level were harvested for gene analysis in sesamoside oral administered mice. PBS was used as a control to sesamoside as it is a solvent of sesamoside. (**A**–**D**): * *p* < 0.05, ** *p* < 0.01, **** *p* < 0.0001 vs. Pacli+PBS with two-way ANOVA followed by Tukey’s multiple comparisons test. (**E**): one-way ANOVA followed by Tukey’s multiple comparison test.

As demonstrated by HPLC (Figure 5), sesamoside, is a component of *P.Radix* extract. Thus, to evaluate whether sesamoside could indeed alleviate the cold and mechanical hypersensitivity induced by paclitaxel, behavioral tests were conducted. Sesamoside was administered to mice intraperitoneally (Figure 6A,B) and orally (Figure 6C,D) at concentration of 5 mg/kg and 7.5 mg/kg. The doses were calculated according to the results obtained by HPLC study: As the *P.Radix* extract consists of 1.5% sesamoside, 5 mg/kg (300 mg/kg × 1.56% ≅ 5 mg/kg) and 7.5 mg/kg (500 mg/kg × 1.56% ≅ 7.5 mg/kg) were used. Behavioral tests were conducted on D15 after paclitaxel injection as in the previous experiment (Figure 2A). Acetone drop and von Frey filament tests were used to assess cold (Figure 6A,C) and mechanical hypersensitivity (Figure 6B,D) in mice, respectively. When sesamoside was injected intraperitoneally, only the 7.5 mg/kg treated group mice showed decreased pain compared to the control (paclitaxel+PBS); however, when sesamoside was given orally, both 5 mg/kg and 7.5 mg/kg sesamoside significantly decreased the cold allodynia. In mechanical allodynia, only 7.5 mg/kg was effective. Subsequently, as the oral administration was more effective, the L4−5 spinal segments of the orally treated mice were sampled, and the gene expression of TRPV1 was analyzed. The result show that spinal TRPV1 gene expression was significantly downregulated in the 7.5 mg/kg but not the 5 mg/kg treated group mice (Figure 6E).

## 4. Discussion

In this study, *P.Radix* extracts significantly alleviated the cold and mechanical hypersensitivities induced by multiple paclitaxel administrations. The effect of *P.Radix* extract was shown to be mediated by spinal TRPV1 as TRPV1, but not TRPM4, and downregulated after *P.Radix* extract administrations. To the best of our knowledge, this is the first study to report the analgesic effect of *P.Radix* on chemotherapy-induced neuropathic pain and to demonstrate its spinal TRPV1 modulating effect.

Chemotherapeutic drugs are known to induce peripheral neuropathy (CIPN) in up to 60% of treated patients [43], and it is known to decrease their quality-of-life and to cause a serious burden on socio-economic level [44]. Paclitaxel is a widely used chemotherapy drug that impairs the mitosis rate of tumor cells by inhibiting the dynamic of microtubules [6]; however, dysesthesia induced by paclitaxel is expressed in abnormal responses to cold and mechanical stimuli in the extremities [45]. As its pathophysiological mechanism, impaired microtubules dynamic [46], axonal degeneration and the demyelination of sensory neurons [47,48], and a change in glial functions have been reported [49]. In the spinal cord, the increased activity of dorsal horn neurons [50] by decreased serotonin release [51] and local γ-Aminobutyric acid (GABA)-induced tonic inhibition [52] or increased TRP channels [53,54] have been suggested as mechanisms.

The TRP channel family consists of a group of non-selective cation channels found in most cell membranes except the mitochondrial membrane and the nucleus. Many excitable and non-excitable tissues express TRP channels, and TRP is involved in sensory signaling for temperature, taste, vision, pressure, and nociception [10]. TRP channels are highly expressed in neurons and are reported to mediate neuropathic pain by synthesizing several inflammatory mediators associated with neurotoxicity and neuroprotection [55]. They contribute to intracellular signal transduction and the transmission of pain stimuli [56,57], and pain therapies targeting the TRP channel have become a promising study.

In this study, multiple paclitaxel injections increased the gene expression of spinal *TRPV1* and *TRPM4* but not *TRPA1*. TRPV1 induced Ca^2+^ and Na^+^ influxes through transmembrane ion channels [58,59] and were localized primarily in small- and medium-diameter neurons, serving as polymodal integrators in muscle, skin, and internal organs [60]. In the spinal cord, it is also expressed in microglia and astrocytes [61]. In a study conducted with paclitaxel, TRPV1 protein was upregulated in the spinal dorsal horn when treated with 8 mg/kg of paclitaxel, as in our study [54]. Furthermore, the spinal injection of AMG9810, a TRPV1 antagonist, significantly suppressed thermal hyperalgesia and mechanical allodynia [54]. Furthermore, in the mice spinal cord, the paclitaxel-induced increase in spinal miniature excitatory post-synaptic current (mEPSC) frequencies decreased after TRPV1 antagonist treatment, demonstrating the involvement of TRPV1 in paclitaxel-induced neuropathic pain [62]. Altogether these results show that TRPV1 receptor expression in the spinal cord contributes to the development and maintenance of paclitaxel-induced neuropathic pain. Although the role of TRPM4 is not well studied, it is known to be activated by high intra-cellular Ca^2+^ concentration and change in voltage [63]. As paclitaxel has been reported to increase the intracellular calcium signaling neuronal calcium sensor 1, a well-known calcium-binding protein [64], the change in spinal *TRPM4* mRNA may be due to the augmented intracellular calcium level following Taxol-based chemotherapy drug injections.

On the contrary to *TRPV1* and *TRPM4*, paclitaxel did not increase *TRPA1* gene expression. TRPA1 is known to respond to a variety of endogenous reactive compounds associated with pathological pain conditions. The large ankyrin repeat region distributed in TRPA1 is known to provide elasticity to the receptor and function as a mechanical stimuli sensor contributing to pain [24,65]. However, some papers have reported the limited role of TRPA1 in pain as reduced mechanical pain was shown in TRPA1 ion channel transgenic mice [66]. Moreover, although TRPA1 are known to co-express in TRPV1-positive neurons and their role in cold and mechanical pain appears to be similar, TRPV1 was demonstrated to play a more critical role in thermal hyperalgesia than TRPA1 in platinum-based chemotherapeutic agent (i.e., cisplatin)-administered rodent [67].

In this study, *P.Radix* extract significantly alleviated the cold and mechanical hypersensitivity induced by multiple paclitaxel injections. This effect was shown to be mediated by spinal TRPV1 but not TRPM4 or TRPA1. The medicinal effects of *P.Radix* have been reported by several papers [68,69]. Furthermore, in a study conducted by Shang et al. [35], the anti-nociceptive and anti-inflammatory effects of *Phlomis umbrosa* Turcz. have been reported. In their study, various animal models of pain and inflammation, such as the acetic acid-induced writhing test, the hot plate test, and the carrageenan-induced paw edema test, were used to assess the effect of the plant extract. Among the three major component of *Phlomis umbrosa* Turcz. (i.e., sesamoside, shanzhiside methylester, and 8-O-acetylshanzhiside methylester), sesamoside was shown to be major component. Accordingly, as it was shown to be in greater quantity in the root than aerial parts of the plants, in this study, the dried root of *Phlomis umbrosa* Turcz. (i.e., *P.Radix*) was used along with sesamoside.

The HPLC analysis showed that the content of sesamoside was about 1.56% in *P.Radix* ethanol extract. Thus, as 300 and 500 mg/kg of *P.Radix* extracts alleviated the cold and mechanical hyperalgesia in mice, 5 and 7.5 mg/kg of sesamosides were chosen as administered doses, respectively. The results showed that only 7.5 mg/kg of sesamoside significantly decreased the pain evoked by paclitaxel when administered intraperitoneally, whereas oral administrations of both 5 and 7.5 mg/kg of sesamoside significantly decreased the cold allodynia; however, in mechanical allodynia, only 7.5 mg/kg was effective as in intraperitoneal injection. Furthermore, the spinal *TRPV1* gene expression was significantly reduced in the 7.5 mg/kg but not the 5 mg/kg sesamoside-treated group. Although the number of studies conducted with sesamoside is small, sesamoside has been previously reported to have an antioxidant effect [70]. However, it should not be excluded that other components of *P.Radix*, such as shanzhiside methylester, 8-O-acetylshanzhiside methylester, and umbroside, may also play an important role in pain.

In conclusion, through this study, we have demonstrated the dose-dependent analgesic effect of *P.Radix* extracts and its spinal TRPV1-modulating effects on paclitaxel-induced neuropathic pain in mice. Paclitaxel injections increased the expression of the *TRPV1* and *TRPM4* genes and protein expressions in the spinal cord; however, *P.Radix* extracts suppressed the increase in spinal *TRPV1* but not *TRPM4*. Sesamoside, a major component of *P.Radix*, significantly reduced both hyperalgesias, suggesting that the effect of *P.Radix* may be due to sesamoside. However, further well-designed studies are needed to better understand the effect of *P.Radix* and sesamoside in chemotherapy-induced neuropathic pain.

## Figures and Tables

**Table 1:** Analytical conditions of HPLC.

Conditions			
Treatment	Sesamoside		
Column	Ymc-Triart C18 Length: 25 cm Diameter: 0.46 cm Particle Size: 5 μm		
Flow rate	1.0 mL/min		
Injection volume	10 μL		
UV detection	234 nm		
Run time	25 min		
Time (min)	%ACN	0.1% Phosphoric acid	Flow (mL/min)
0	5	95	1.0
15	40	60	1.0
16	100	0	1.0
19	100	0	1.0
20	5	95	1.0
25	5	95	1.0

## Data Availability

All the data supporting the conclusions of this study are included in the manuscript.
